# Evidence that the aesthetic preference for Hogarth’s Line of Beauty is an evolutionary by-product

**DOI:** 10.1038/s41598-023-31175-w

**Published:** 2023-03-13

**Authors:** Ronald Hübner, David M. G. Lewis, Laith Al-Shawaf, Ayten Yesim Semchenko, Jonathon Flores

**Affiliations:** 1 Department of Psychology, University of Konstanz, D-78457 Konstanz, Germany; 2 School of Psychology, Murdoch University, Murdoch, Australia; 3 Centre for Healthy Ageing, Health Futures Institute, Murdoch University, Murdoch, Australia; 4 Psychology Department, University of Colorado-Colorado Springs, Colorado Springs, USA; 5 Faculty of Science, Charles University, Prague, Czech Republic; 6 Houston Christian University, Houston, USA

**Keywords:** Evolution, Psychology

## Abstract

In 1753, artist William Hogarth declared a specific S-shaped line to be *the* ‘Line of Beauty’ (LoB). Hogarth’s assertion has had a profound impact on diverse fields over the past two and a half centuries. However, only one recent (2022) study has investigated whether Hogarth’s assertion accurately captures humans’ actual aesthetic preferences, and no research has explored *why* people find the LoB beautiful. We conducted two studies testing the hypothesis that the LoB’s perceived beauty is an incidental by-product of cognitive systems that evolved to attend to fitness-relevant morphological features in people. In Study 1, we replicated the finding that female bodies whose lumbar curvature approximates the biomechanical optimum for dealing with the exigencies of pregnancy are rated as more attractive. In Study 2, we found that abstract lines extracted from these bodies were perceived as more beautiful than other lines. These results suggest that the preference for Hogarth’s LoB is an incidental by-product of psychological mechanisms that evolved for other purposes. More broadly, these findings suggest that an evolutionary psychological approach – in particular the concept of evolutionary by-product – may be useful for understanding, explaining, and predicting people’s aesthetic preferences for certain abstract symbols, which otherwise might seem arbitrary and inexplicable.

## Introduction

“…[S]trictly speaking, there is but one precise line, properly to be called the Line of Beauty, which … is number 4*”* (p. 49, Hogarth, 1753)*.*

**Figure 1:**
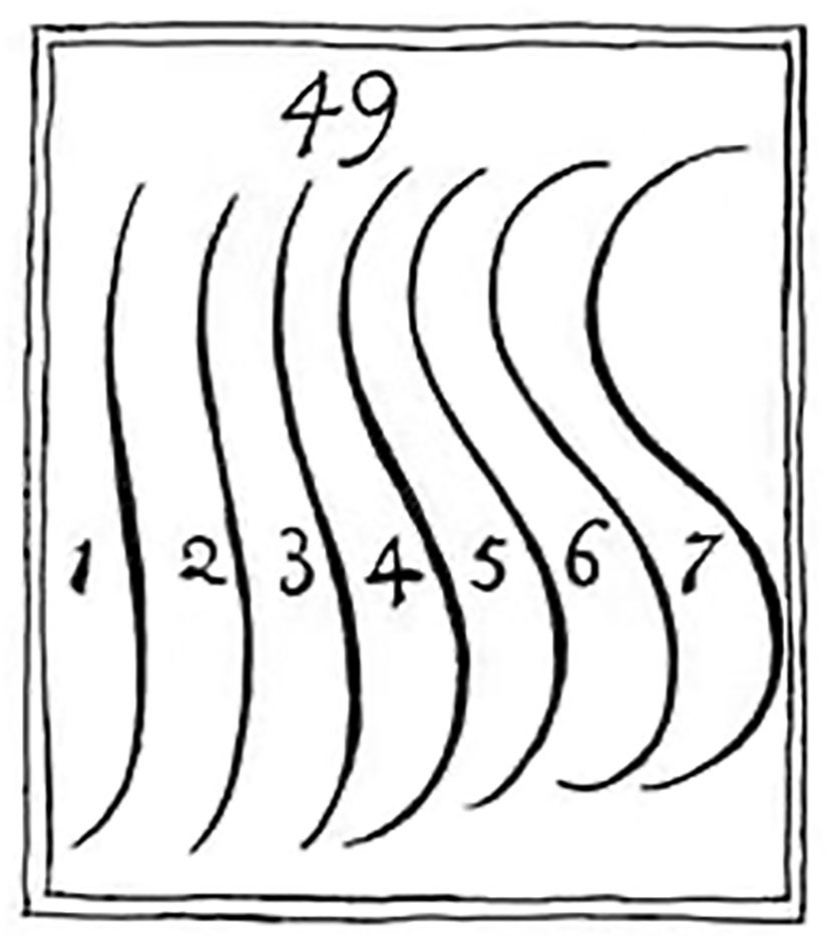
Illustration 49 from Plate I in William Hogarth (1753).

In his 1753 book ‘The Analysis of Beauty’^1^, British artist, printmaker, and theorist William Hogarth proposed that wavy lines are more beautiful than straight or curved ones, and that this especially holds for S-shaped lines, as shown in Fig. 1. Hogarth further asserted that line number 4 specifically was the most beautiful and called it the “Line of Beauty” (LoB). Hogarth’s LoB has been considered an ideal and has had a profound and enduring effect on diverse fields, from landscaping^2^ and furniture design^3^ to calligraphy^4^, hairdressing^5^, poster (propaganda) composition^6^, and dance^7,8^.

Remarkably, this influence of the LoB has been based largely on the *assumption* that it is the most beautiful − prior to 2022, there were no empirical data whatsoever demonstrating that people actually perceive Hogarth’s LoB to be the most aesthetically pleasing. Hübner and Ufken^9^ were the first to test this assumption, and found that line 4 (i.e. the LoB) was perceived to be very attractive.

Hübner and Ufken’s^9^ results are valuable because they contribute the first empirical tests of this presumed standard of beauty that has been highly influential in art, design, and other fields. However, two important questions remain unresolved. First, no work has identified the specific characteristics that influence a line’s beauty. Hogarth’s account that lines are less beautiful when they “straighten becoming mean and poor” or “bulge too much in their curvature, making them gross and clumsy” is underspecified and subjective. Hübner and Ufken^9^ linked perceptions of beauty to objective features of curves, but they only tested the relationship between beauty and these objective features for Hogarth’s lines, which raises the question of whether their results obtain for other lines, or for more ecologically valid stimuli.

Second, identifying the objective geometric properties that increase the perceived beauty of lines is informative, but it leaves a deeper question unaddressed: *why* do people perceive lines with those properties to be the most beautiful? Here, we advance and test the novel hypothesis that people’s perceptions of the beauty of wavy lines are an incidental by-product of cognitive systems that evolved to attend and respond to reproductive fitness-relevant cues in conspecifics (for discussion of what an incidental by-product is, see Ref.^10–12^).

### Line of Beauty and body shape

**Figure 2:**
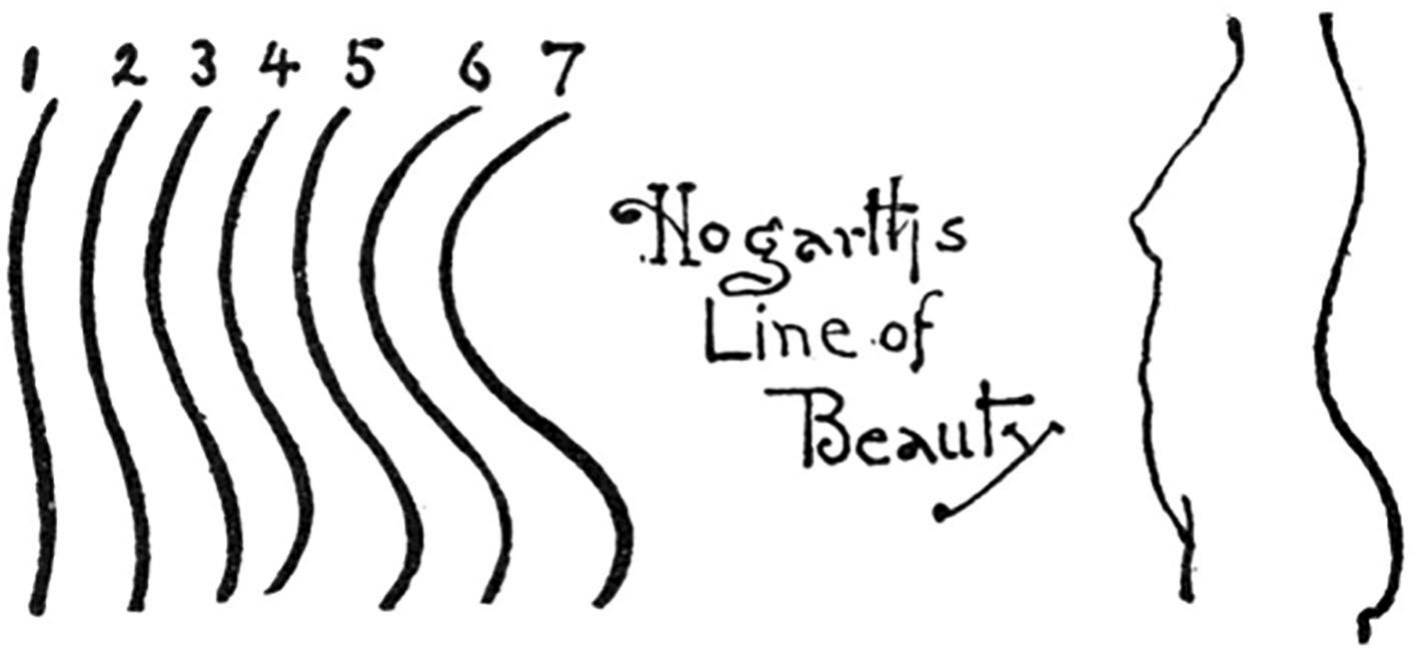
Part of the figure on page 94 in Poore^13^.

Hogarth himself proposed that the attractiveness of specific wavy lines (e.g. the LoB) might relate to a woman’s body, in particular its back profile^1^. This idea was further developed and specified in the early 1900s by the American painter and author Henry Rankin Poore, who explicitly related Hogarth’s lines to the lumbar region of a woman’s body in his book *Pictorial composition and the critical judgement of pictures*^13^. In one of Poore’s figures (Fig. 2), he roughly reproduced Hogarth’s lines and displayed the profile of a woman’s torso (facing left) in such a way that the upper arc corresponds to the curve formed between the thoracic spine and the sacrum and the lower arc to the buttocks (see Fig. 2).

### Lumbar curvature: an evolutionarily relevant feature

The specific shape of the human back reflects evolutionary selection pressures on the spine to manage loads during upright walking. However, the physics of the load-bearing requirement differs between men and women: because gestation occurs exclusively among females in the hominin lineage, ancestral women (but not ancestral men) faced the challenge of a forward-shifted center of mass during pregnancy (see Ref.^14,15^). During human evolution, if ancestral women were unable to move their center of mass back over the hips during pregnancy, this would have dramatically increased torque on the hips, resulting in muscular fatigue and increased risk of back injury, thereby critically reducing foraging efficiency. Consequently, selection favored a morphological adaptation in women’s third-to-last lumbar vertebra: wedging, which translates to a more acute angle formed between the sacrum and thoracic spine, and is externally visible in the degree of women’s lumbar curvature. Based on evidence from the medical orthopedic literature, Lewis, et al.^14^ operationalized the optimal degree of lumbar curvature (with respect to sustaining a fetal load without suffering back injury) as approximately 45.5°, and hypothesized that evolution by natural selection should have shaped attractiveness-assessment mechanisms in the human mind to perceive approximately this angle of lumbar curvature as more beautiful than other angles.

### The current studies

In the present research, we investigated whether the lumbar curvature hypothesis might be able to explain why Hogarth’s LoB is perceived as most beautiful. This entailed collecting different forms of evidence. This included (1) beauty ratings of photorealistic images of female bodies, (2) extracting abstract S-shaped lines from these bodies (henceforth “body lines”), and (3) assessing which of Hogarth’s lines most closely corresponds to the body line (BL) with the proposed evolutionarily optimal degree of lumbar curvature.

If the lumbar curvature hypothesis is correct, both as an adaptation hypothesis about the perceived beauty of women’s bodies and as a by-product explanation for the perceived beauty of specific wavy lines, then we should observe three outcomes: (1) women with the proposed optimal curvature will be perceived as most attractive, (2) the abstract BLs derived from women with the proposed optimal curvature will be perceived as most attractive, and (3) the LoB will be most similar in objective properties to these most attractive BLs.

## Study 1

In Study 1 we had participants assess the beauty of photorealistic images of female bodies that varied in their degree of lumbar curvature. This enabled us to test whether Lewis et al.′s^14^ findings replicated with respect to the relationship between women’s lumbar curvature and perceptions of their beauty.

### Method

#### Participants

Nine hundred eighteen participants (467 men) participated in the online study conducted in Czech. The majority of the sample was Czech, with a Slovak minority, as well as a small number of other Czech speakers (e.g. Russians). Due to an error in the online survey, the specific ages of participants 40 years of age or older (*N* = 189) were not successfully recorded; participants whose age was below 40 had a mean age of 30.0 (*SD* = 5.69) years. This study was approved by the Institutional Review Board of Charles University, Czech Republic (approval #2020/13). In accordance with the Declaration of Helsinki, all participants provided informed consent. Because data were collected online, participants indicated their consent electronically in lieu of providing a written signature.

#### Stimuli

**Figure 3:**
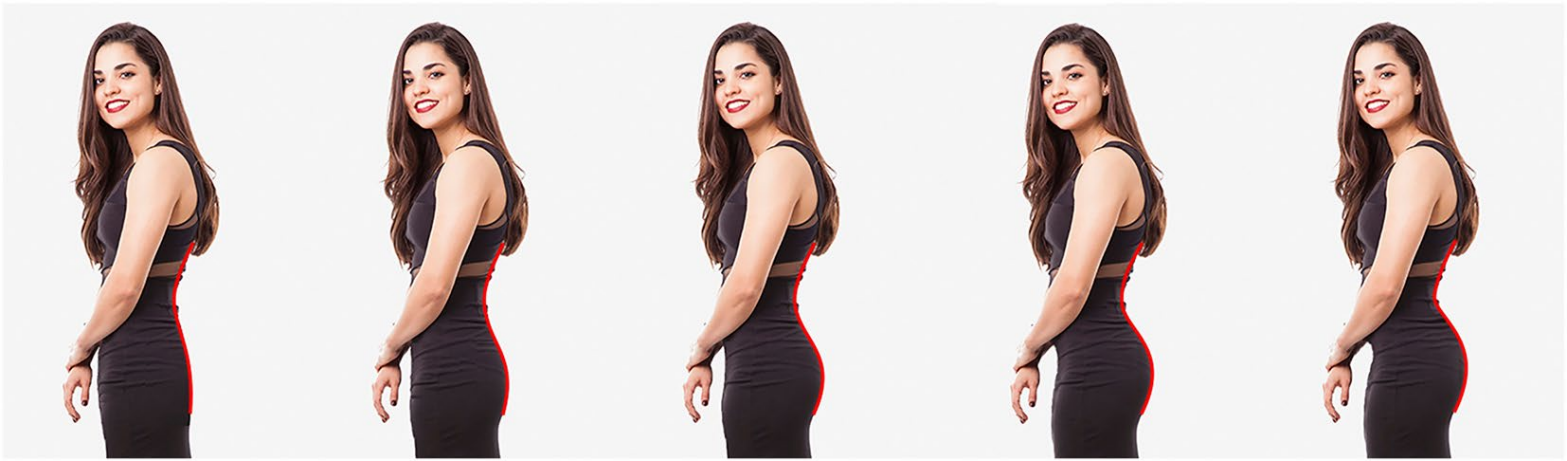
Photorealistic stimuli (morphs) depicting female characters varying in lumbar curvature. The red lines (not shown to participants in Study 1) represent the corresponding body lines used in Study 2. The photorealistic stimuli were reproduced with permission from Lewis et al.^16^.

We used the five photorealistic images from Lewis et al.^16^. These stimuli (reproduced with permission in Fig. 3) varied in their degree of lumbar curvature; from left to right, the morphs have angles of lumbar curvature of 32°, 39°, 44°, 49°, and 54°, respectively. These angles all fall within the naturally occurring range of lumbar curvature in human populations (e.g. see Ref.^17^).

#### Procedure

As part of a longer survey on perceptions of beauty, participants were presented with each of the five body morphs, one by one, in random order, with order randomized anew for each participant. For each morph, participants were asked to rate the woman’s attractiveness (1) as a short-term mate and (2) as a long-term mate (0 = extremely unattractive, 10 = extremely attractive).

### Results

For the purposes of the current paper, we created a single, composite measure of beauty by averaging the short-term and long-term attractiveness ratings, which were highly correlated for all five morphs (Spearman’s *r* > 0.68 for all morphs, *p* < 10^–129^ for all morphs). These beauty ratings were entered as the dependent variable in a two-way ANOVA with the within-participant factor Morph (1 to 5) and the between-participants factor participant Gender (woman, man) as predictor variables.

**Figure 4:**
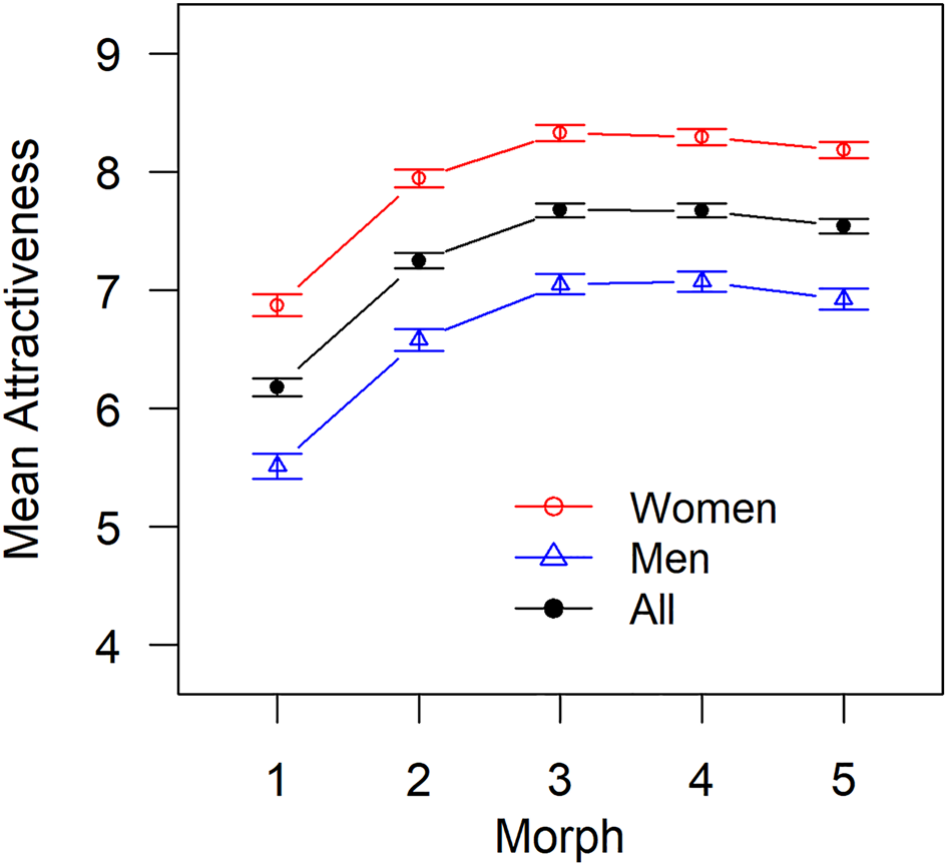
Mean attractiveness ratings of the five morphs in Study 1.

These analyses revealed significant effects of Morph, *F*(2.57, 2353) = 372, *p* < 0.001, GES = 0.089, and Gender, *F*(1, 916) = 155, *p* < 0.001, GES = 0.114. The Morph × Gender interaction was not significant, *F*(2.57, 2353) = 372, *p* = 0.436. Collectively, these results show that (1) specific morphs were perceived as more beautiful than others, and (2) this effect did not differ between men and women (see Fig. 4), although (3) women perceived the stimuli to be more beautiful, on average, than men (7.92 vs. 6.63).

To probe finding 1, i.e. that specific morphs were perceived as more beautiful than others, we conducted post hoc comparisons (Bonferroni-adjusted *t* tests) between all pairs of morphs to identify which specific morphs were perceived as more beautiful than others. These analyses revealed that morphs 3 and 4 were significantly more attractive than all other morphs (*p*_adj_ < 0.01 for all comparisons), and that morph 1, whose lumbar curvature was furthest from the proposed optimum, was less attractive than all other morphs (*p*_adj_ < 0.001 for all comparisons).

### Discussion

These results replicate Lewis et al.'s^14^ key findings: the female morphs whose lumbar curvature was closest to the proposed fetal load optimum were perceived as more beautiful than morphs whose lumbar curvature deviated further from this proposed optimum. This replication finding set the stage for the test of our central by-product hypothesis: are abstract lines derived from evolutionarily optimal human bodies perceived to be more beautiful than lines derived from other bodies? And if so, might this provide an ultimate explanation for why people find Hogarth’s Line of Beauty aesthetically pleasing?

## Study 2

**Figure 5:**
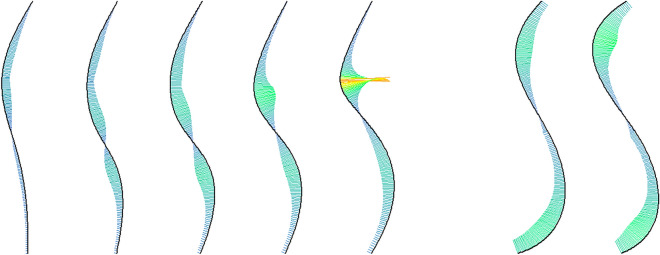
The five body lines (5 left lines) and their curvature. The curvature at each point along each line is indicated by the length (scaled for good visibility) and color (redundant coding) of the straight lines orthogonal to the tangent at that point. For comparison, Hogarth’s lines number 4 and 5 are also shown at the right. Curvature was computed with the R Package ‘knotR’^18^.

In Study 2, we used Hübner and Ufken’s^9^ method for modelling the Hogarth lines – namely, the fitting of two concatenated cubic Beziér curves to each morph – to extract body lines (BLs), shown in red in Fig. 3. This enabled us to compute specific properties of the lines (e.g. their curvature at each point; Fig. 5), which enabled an objective quantitative comparison between Hogarth’s lines and the body lines.

If the by-product hypothesis of people’s aesthetic preferences for Hogarth’s LoB is correct, then we should expect to observe the following. First, the BL derived from the morph whose lumbar curvature is closest to the proposed optimum should be perceived to be the most beautiful. Second, Hogarth’s LoB (HL 4) should be more similar to this body line than to any other body line.

**Figure 6:**
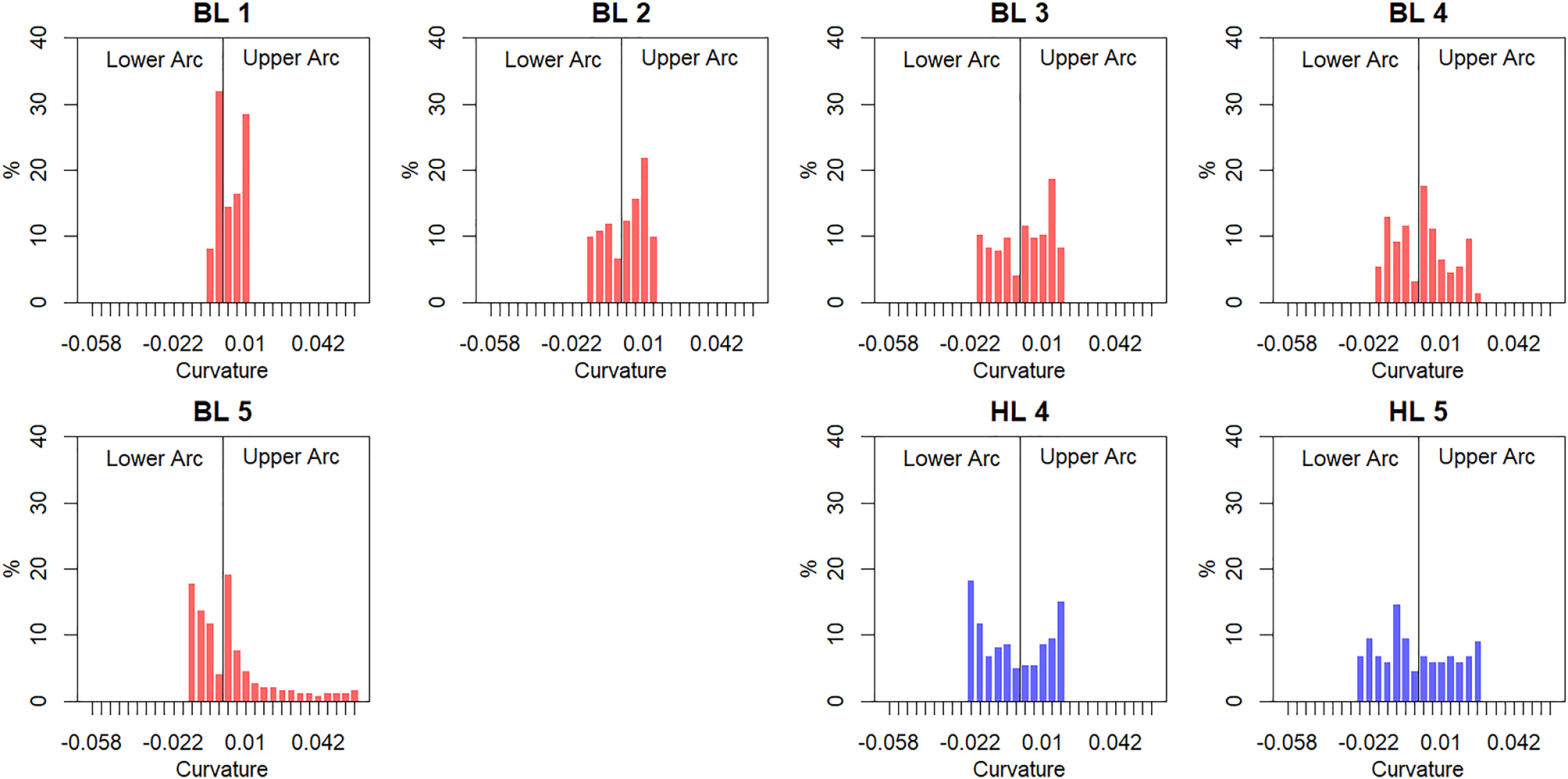
Curvature histograms (scaled to percent) of the five body lines (in red) and Hogarth lines 4 and 5 (in blue).

There were multiple possible ways in which we could operationalize similarity between Hogarth’s lines and the body lines. One possibility would be to use an index of mean absolute curvature, as Hübner and Ufken^9^ did to predict the beauty ratings of the HLs. However, this simple measure is inadequate in the present context, as there are an infinite number of (very different) lines with the same mean curvature. An alternative and more appropriate operationalization of the similarity between lines is their Histogram Difference: the sum of the absolute differences between the curvature histograms of two lines. This comprehensive index of line similarity revealed that BL 3 was most similar to HL 4—that is, the Line of Beauty (see Fig. 6, Table 1).

**Table 1:** Sum of absolute differences between the curvature histograms of the five body lines (BL) and Hogarth’s lines (HL) 4 and 5, respectively.

	BL 1	BL 2	BL 3	BL 4	BL 5
HL 4	290	181	**111**	178	232
HL 5	301	194	156	**137**	187

The bold values indicate the smallest difference.

The finding that HL 4 – Hogarth’s declared Line of Beauty – most closely corresponded to Body Line 3 suggested that the by-product hypothesis might indeed provide insight into people’s aesthetics preferences for the LoB. The female morph from whom Body Line 3 was extracted was (i) the morph with the lumbar curvature closest to the proposed evolutionary optimum and (ii) was perceived as most attractive in Study 1. This match between Hogarth’s Line 4 and Body Line 3 is consistent with the notion that people’s aesthetic preferences for specific curvy lines could be an incidental by-product of preferences for specific lumbar curves. Nonetheless, the crucial next step was to empirically test the perceived beauty of the BLs to assess whether BL 3 was found to be the most attractive BL.

### Method

#### Participants

Ninety-eight participants (*M*_age_ = 28.2 years, *SD*_age_ = 8.46, 33 men) participated in the online study in German. The majority of the sample were students at the University of Konstanz, Germany. The study was conducted in accordance with the ethical guidelines of the University of Konstanz, and the Declaration of Helsinki (1964) and its later amendments. Participants were informed of their right to quit the study at any time without reprisal, and their informed consent was obtained by check-marking a box before the study began.

#### Materials and procedure

As part of a larger online study on perceptions of beauty, participants completed a line-rating task in which they were presented with, and asked to rate the beauty of, each of the five BLs individually. Each participant was randomly assigned to either view the original BLs (i.e. left-facing) or their mirror-symmetric (i.e. right-facing) versions. Each BL was presented at the center of a screen-centered empty white canvas (500 × 500 px) on a grey background and had a height of 300 px. For each line, the participant was asked to rate the line’s beauty on a visual analogue scale (1 = not beautiful at all, 100 = very beautiful).

### Results

**Figure 7:**
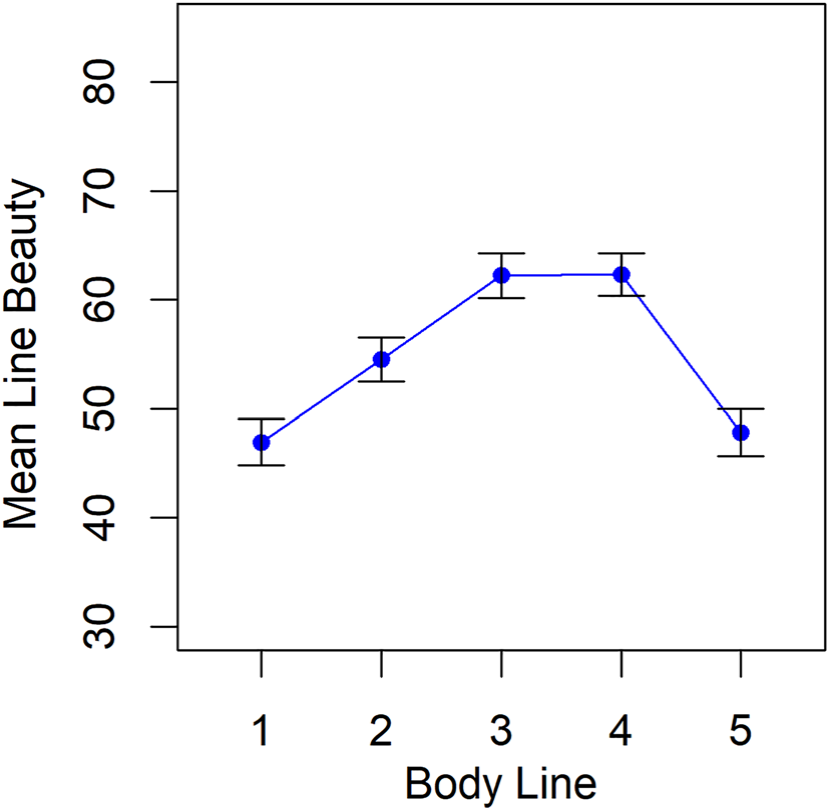
Mean attractiveness ratings of the five body lines in Study 2.

The beauty ratings were entered as the dependent variable in a 5 × 2 × 2 ANOVA with the within-participant factor Body Line (1 to 5), and the between-participants factors Direction (left, right), and participant Gender (man, woman). The ANOVA revealed that neither Direction (*p* = 0.177) nor Gender (*p* = 0.305) had a significant effect. Only the factor Body Line was significant, *F*(4, 376) = 20.8, *p* < 0.001, GES = 0.096, (Fig. 7).

To probe this effect of Body Line – which indicated that some lines were perceived as more beautiful than others – we conducted post hoc comparisons (Bonferroni-adjusted *t* tests) between all pairs of BLs. This enabled us to identify which specific BLs were perceived as more beautiful than others. All pairwise comparisons were significant, except between lines 1 and 5, *t*(97) = 0.341, *p*_adj_ = 0.734, and between lines 3 and 4, *t*(97) = 0.05, *p*_adj_ = 0.956. Most importantly, the analyses revealed that BLs 3 and 4 – which were derived from the morphs with the lumbar curvature angles closest to the proposed evolutionary optimum – were perceived to be more beautiful than all other BLs.

### Discussion

Study 2 data show that people have clear aesthetic preferences with respect to the abstract lines (which, unbeknownst to the participants, were derived from different female bodies), and that the two lines derived from the bodies whose lumbar curvature was closest to the evolutionary optimum were perceived as the most beautiful. Furthermore, the curvature histogram of HL 4 (i.e. Hogarth’s LoB) was most similar to that of BL 3 — the body line whose lumbar curvature was *the* closest to the proposed evolutionary optimum and which was perceived as most beautiful (together with BL 4).

These results are consistent with the hypothesis that people’s perceptions of the beauty of abstract S-shaped lines are an incidental by-product of psychological mechanisms that evolved to respond to another cue: the evolutionary relevant cue of lumbar curvature.

## General discussion

In the current research, we investigated why specific lines are viewed as more attractive than others. Hogarth^1^ famously proclaimed that wavy lines are more beautiful than straight ones, presented seven S-shaped lines of increasing curvature, and declared line number 4 to be the Line of Beauty. This line, and this assertion, have been influential in numerous fields of human design and behavior, including art, propaganda posters, and dance. Hübner and Ufken^9^ were the first to empirically confirm Hogarth’s assertion; they found that people find this Hogarth line (together with Hogarth line number 5) to be the most beautiful. However, no existing research or writing addresses the ultimate question of *why* people perceive those lines to be more beautiful than others?

Our studies here may provide a preliminary answer to this question. Our results are consistent with the idea that people’s aesthetic preference for the Line of Beauty is an incidental by-product of cognitive systems that evolved to respond to a different cue: the reproductive fitness-relevant cue of lumbar curvature. We found that (1) people had clear aesthetic preferences with respect to the abstract lines (derived from female bodies, unbeknownst to participants), (2) the two lines that were derived from female bodies whose lumbar curvatures were closest to the proposed evolutionary optimum were perceived as the most beautiful, and (3) the curvature histogram of HL 4 (i.e. Hogarth’s Line of Beauty) was most similar to that of Body Line 3 — the line derived from the female body whose lumbar curvature was the closest to the proposed evolutionary optimum and which was perceived as the most beautiful (together with BL 4). In short, the results from the current two studies are multifaceted, and each facet is consistent with the proposed by-product account of people’s preferences for S-shaped curves.

One reviewer raised the interesting question whether the differences in beauty between Hogarth’s lines are only quantitative or whether at least some lines also differ qualitatively. As mentioned, in our earlier research^9^, we showed that there is a quantitative relationship between curvature and beauty in the seven lines. Since we assessed beauty using ratings, we cannot say whether there are also qualitative differences in beauty among the lines. However, given the data in the present study, it is conceivable that the beauty of certain lines, e.g. Hogarth’s line number 4, also differs qualitatively from that of the other lines. For instance, it could be that viewing this line strongly activates memory representations of a beautiful female body and associated positive feelings, and that these activated memory structures then also make a qualitative difference in the perceived beauty of the line, compared to the other Hogarth lines. Future research must show to what extent this is really the case.

### Limitations and future directions

The current studies have additional limitations that point toward other important future directions. One question that the current studies are incapable of answering is whether the observed preferences for the specific body morphs and abstract lines derived from them are uniform across cultures. Ultimately, this question can only be answered by further empirical research. However, here we describe how an evolutionary psychological perspective would approach the issue of cross-cultural variability or uniformity in standards of beauty.

Despite the common misperception that an evolutionary approach posits fixed preferences, it does not: evolutionary psychology proposes species-typical information-processing systems and emphasizes that the output of these systems (e.g. preferences) should vary in response to environmental inputs that shift the costs and benefits of the possible outputs from those systems (see Ref.^19^ and Ref.^20^ for reviews; see also Ref.^20–22^).

This evolutionary approach generates two key a priori hypotheses about the information-processing systems responsible for regulating perceptions of attractiveness. First, natural selection should have shaped these attractiveness-assessment mechanisms to attend to morphological traits in potential mates that had *fitness value* (i.e. that were ancestrally predictive of positive or negative fitness consequences of mating with that individual). Second, if the fitness value of a particular trait varied across contexts, then selection should have shaped attractiveness-assessment mechanisms to be sensitive to those contextual variables. Several different lines of research provide evidence that this is how humans’ attractiveness-assessment mechanisms operate. Here, we provide two examples.

First, in environments characterized by the threat of food shortages, higher levels of body fat stores have greater fitness value than they do in resource-abundant conditions. An evolutionary psychological perspective would therefore lead us to expect human attractiveness-assessment mechanisms to place greater value on body fat stores in food-scarce environments compared to food-abundant environments (e.g. see Ref.^23^, see also Ref.^20^). Consistent with this idea, evidence suggests that people from foraging societies, compared to people from WEIRD (Western, Educated, Industrialized, Rich and Democratic) societies, prefer mates who have higher levels of body fat (see Ref.^19^ and Ref.^24^; for reviews see ref.^20–22,25^).

Second, androgen-linked traits, such as men’s facial masculinity, are hypothesized to cue immunocompetence and the siring of healthy and robust offspring—but these traits are also linked with lower levels of paternal investment. The precise calculus of the trade-off between producing highly immunocompetent offspring and forgoing paternal investment depends on environment: the benefits of producing highly immunocompetent offspring are greater in environments characterized by greater prevalence of communicable diseases. Consequently, we might expect selection to have shaped women’s attractiveness-assessment mechanisms to place greater value on men’s facial masculinity in environments that have higher levels of disease. Consistent with this hypothesis, women from nations with greater impact of communicable disease, higher mortality rates, and lower life expectancy prefer higher levels of facial masculinity in potential mates than do women from cultures characterized by lower levels of survival threat (see Debruine et al.^26^, who tested this hypothesis across 30 different countries). These examples illustrate how an evolutionary approach actively predicts (and has led to the discovery of) cross-cultural variation in preferences.

However, an evolutionary approach does not always predict cross-cultural variation in the preferred levels of a trait; it predicts cross-cultural variation specifically when the fitness value of a trait varies across cultures. In the case of women’s lumbar curvature, we would not necessarily expect the optimal level to vary across environments. This is because the vertebral wedging underlying women’s lumbar curvature evolved to solve the adaptive problem of a forward-shifted center-of-mass during pregnancy. This adaptive problem is invariant across environments; the problem of a forward-shifted center-of-mass during pregnancy holds equally in all environments. Because of the likely invariance of this adaptive problem, there may not be principled theoretical reasons to expect selection to have shaped attractiveness-assessment mechanisms to produce variable preferences for women’s lumbar curvature across different cultures. Consistent with this idea, available data, which have been collected from people from three different continents, suggest that the preferred level of female lumbar curvature is uniform across nations and does not vary as a function of other contextual variables such as mating context (e.g. short-term vs. long-term) that are known to shift the fitness value of other traits (see Ref.^14,27,28^; see also Ref.^29^).

More data are needed to determine whether there is cross-cultural variation in preferences for female lumbar curvature and in the specific abstract lines that people find most beautiful. Here, we emphasize that cross-cultural variation in standards of beauty is not evidence against an evolutionary perspective. To the contrary, as elaborated above, an evolutionary approach frequently predicts cultural variation in standards of attractiveness and perceptions of beauty. Nonetheless, more data from a more diverse set of cultures are needed to provide an empirical answer to questions about variability in the preferences documented in the current studies.

A related limitation is that the current studies cannot directly address whether perceptions of beauty have changed over time. The same evolutionary model described above may be useful for thinking about this issue as well. If the fitness value of a given trait varied across contexts, selection should have shaped attractiveness-assessment mechanisms to be sensitive to contextual variables that shift the fitness value of that trait. However, if the fitness value of a trait did not vary across contexts (e.g. across different timepoints in human history), we might not expect the preferred level of that trait to vary across different eras of human history. Because there is no reason to think that the adaptive problem of a forward-shifted center-of-mass during pregnancy has varied at any point during human evolution, we should not necessarily expect preferences for women’s lumbar curvature to vary across time periods. The current study data are at least circumstantially consistent with this notion: our participants’ perceptions of beauty in the twenty-first century align closely with a standard of beauty proposed in 1753, which was a very different historical era: plants had not yet been scientifically classified, there were no independent countries in the “New World”, and it would be another 100 years before Darwin would publish his theory of evolution by natural selection. Nonetheless, because the studies presented in this manuscript are cross-sectional and taken during a specific snapshot in time, they cannot provide a definitive answer to the question about whether the observed preferences have varied across time periods. This therefore represents a study limitation that awaits further research.

## Conclusions

The findings from the current two studies highlight the value of an evolutionary psychological approach – in particular the conceptual tool of *incidental by-products* – for understanding aesthetics and other domains of psychology to which evolution might not immediately appear relevant. By-product hypotheses may be indispensable for understanding how humans’ evolved psychology can produce effects that at first blush seem irrelevant to evolution—such as preferences for specific types of abstract lines. By-product hypotheses show how these phenomena may not have an evolved function and may not solve any adaptive problem but can nonetheless be illuminated by an evolutionary approach to the human mind. Outside of an evolutionary psychological framework, these preferences might seem arbitrary, and without the conceptual tool of evolutionary by-products, they might otherwise remain unexplained. It is our hope that future research makes similar use of this invaluable but scarcely used conceptual tool to generate new discoveries and identify the deeper origins of contemporary psychological phenomena that currently elude explanation.

## Data Availability

Study data are available at https://osf.io/qdse6/?view_only=73b3b9277b0048f0ae308339226adc8b.
